# Retina-Based Pipe-Like Object Tracking Implemented Through Spiking Neural Network on a Snake Robot

**DOI:** 10.3389/fnbot.2019.00029

**Published:** 2019-05-29

**Authors:** Zhuangyi Jiang, Zhenshan Bing, Kai Huang, Alois Knoll

**Affiliations:** ^1^Chair of Robotics, Artificial Intelligence and Real-time Systems, Department of Informatics, Technical University of Munich, Munich, Germany; ^2^Department of Data and Computer Science, Sun Yat-Sen University, Guangzhou, China

**Keywords:** neuromorphic vision, spiking neural network, snake robot, Hough transform, target tracking

## Abstract

Vision based-target tracking ability is crucial to bio-inspired snake robots for exploring unknown environments. However, it is difficult for the traditional vision modules of snake robots to overcome the image blur resulting from periodic swings. A promising approach is to use a neuromorphic vision sensor (NVS), which mimics the biological retina to detect a target at a higher temporal frequency and in a wider dynamic range. In this study, an NVS and a spiking neural network (SNN) were performed on a snake robot for the first time to achieve pipe-like object tracking. An SNN based on Hough Transform was designed to detect a target with an asynchronous event stream fed by the NVS. Combining the state of snake motion analyzed by the joint position sensors, a tracking framework was proposed. The experimental results obtained from the simulator demonstrated the validity of our framework and the autonomous locomotion ability of our snake robot. Comparing the performances of the SNN model on CPUs and on GPUs, respectively, the SNN model showed the best performance on a GPU under a simplified and synchronous update rule while it possessed higher precision on a CPU in an asynchronous way.

## 1. Introduction

Target tracking performed on mobile robots, such as bio-inspired snake robots, remains a challenging research topic. Specifically, when using visual approaches based on the conventional vision sensor which has a rigid connection to a mobile robot, there are mainly two challenges: (1) The primary issue is how to overcome the image blur resulting from the fast motion and the unpredictable tremble of the robot. Meanwhile, if there was no change in a scene, the traditional camera with a fixed frame rate would bring a large quantity of redundant data, which constraints the design and application of real-time tracking approaches. (2) Another issue is that the relative position of the target cannot be obtained fast and precisely from the sensors assembled on the robot, including IMU sensors, vision sensors, and time-of-flight sensors. In addition, owing to the limitation of space and weight of the real snake robot, it is usually difficult to utilize more sensors with higher precision and larger volume or stereo vision sensors for gaining depth information.

There have been extensive articles aiming to solve the aforementioned problems in visual target tracking on robots. A natural solution for tracking on blurred image sequence is to first perform deblurring and then a apply tracking algorithm on the deblurred sequence. An improved method is directly tracking the target without deblurring (Jin et al., 2005). By generating blur templates of the target from blur-free frames, the target is represented by a sparse matrix and tracked by a particle filter (Wu et al., 2011; Ma et al., 2016). Although these frameworks are blur-tolerant, they are still time-consuming. An alternative approach performed on mobile robots is tracking objects in special color. Hu et al. (2009) designed a vision-based autonomous robotic fish and implemented red-ball tracking. However, this method cannot be used in a complex environment or for objects with low color contrast. Recently, researchers have attempted various new types of vision sensors in target tracking, such as structured light sensors (Ponte et al., 2014) and neuromorphic vision sensors (NVS) (Schraml et al., 2010; Glover and Bartolozzi, 2016; Liu et al., 2016; Moeys et al., 2016; Seifozzakerini et al., 2016).

Tracking by using the neuromorphic vision sensors has become a promising solution. The NVS, typically the Dynamic Vision Sensor (DVS) (Lichtsteiner et al., 2008), mimics the biological retina to generate spikes in the order of microseconds in response to the pixel-level changes of brightness caused by motion. An output event (also named as a spike) of the DVS carries three kinds of information, including the timestamp *t* when the event occurred, the pixel coordinate (*x, y*), and the polarity *p* that represents the trend of the brightness change. The polarity 1 represents increasing brightness, while the polarity –1 means the brightness is decreasing. NVSs offer significant advantages over standard frame-based cameras, namely a very high dynamic range, no motion blur, and a latency in the order of microseconds (Gehrig et al., 2018). Hence, the NVS is suitable for working under bad light conditions and on high-speed mobile platforms. There has been substantial research showing the advantages of using a DVS camera in various vision tasks, such as high-speed target tracking (Drazen et al., 2011; Mueggler et al., 2014; Lagorce et al., 2015), object recognition (Kheradpisheh et al., 2018), and visual odometry (Kueng et al., 2016; Rebecq et al., 2017). Moreover, due the fact that a pixel of an NVS is a silicon retinal neuron and an event is a unit impulse with polarity, the asynchronous event train generated by an NVS can be directly fed into Spiking Neural Networks (SNNs) as input spikes for implementing target detecting and tracking in a faster and more brain-like way.

The wheel-less snake robot (Wright et al., 2007) is a kind of typical bio-inspired mobile robot, which is composed of many modules alternately connected in vertical and horizontal planes. Its abundant degrees of freedom help it achieve various three-dimensional gaits, such as rolling, side-winding and slithering. The slithering gait is a forward locomotion gait where the biological snakes use undulations to push their bodies forward (Hu and Shelley, 2012). Under this gait, the snake head can still remain stable to locate the moving direction of the quarry or the natural enemies. Similarly, the wheel-less snake robot is able to move and look forward under a slithering gait and achieve target tracking (Bing et al., 2017).

In this work, we presented a pipe-like object detecting and autonomous tracking framework, which was performed on our wheel-less snake robot with a monocular DVS camera by applying a spiking neural network which is inspired by the Hough transform (Wiesmann et al., 2012; Seifozzakerini et al., 2016). First, we achieved line detection for a standing pipe and circle detection for a lying pipe on the snake robot in the Neurorobotics Platform (NRP). The fixed connections between the input neurons corresponding to pixels of DVS and neurons representing the points in parameter space were created according to the principle of the Hough transform. Secondly, a depth estimation method based on a monocular DVS was proposed to estimate the pose of the snake robot and the relative position of the target pipe by the change of object size. Thirdly, an adaptive tracking strategy which generates a series of control signals of turning left or turning right was adopted to implement real-time tracking. Finally, target tracking experiments were conducted on the wheel-less snake robot modeled in V-REP and in NRP, respectively; and our SNN model was evaluated on CPUs and on GPUs, respectively.

This paper is based on our previous work (Jiang et al., 2017), which we extend in several ways:

- Besides V-REP, we validated our tracking framework in another simulator - NRP.
- We extend the range of shapes to detect and track so that the snake robot can track a target pipe in various views. We not only detected and tracked the standing pipe (shape in lines) but also the lying pipe (shape in circle).
- We revised conditions applied in detecting the standing pipe, which is more biologically plausible.
- GPUs were used as accelerators to speed up object detecting on the SNN.

The rest of the paper is organized as follows. In section 2, we describe the proposed tracking framework, including the overview, the model of spiking neural networks for detecting and the relative position estimation algorithm. In section 3, we show and discuss the results of experiments conducted on a wheel-less snake robot. The conclusions are drawn in section 4.

## 2. Methodology

### 2.1. Tracking Framework

**Figure 1:**
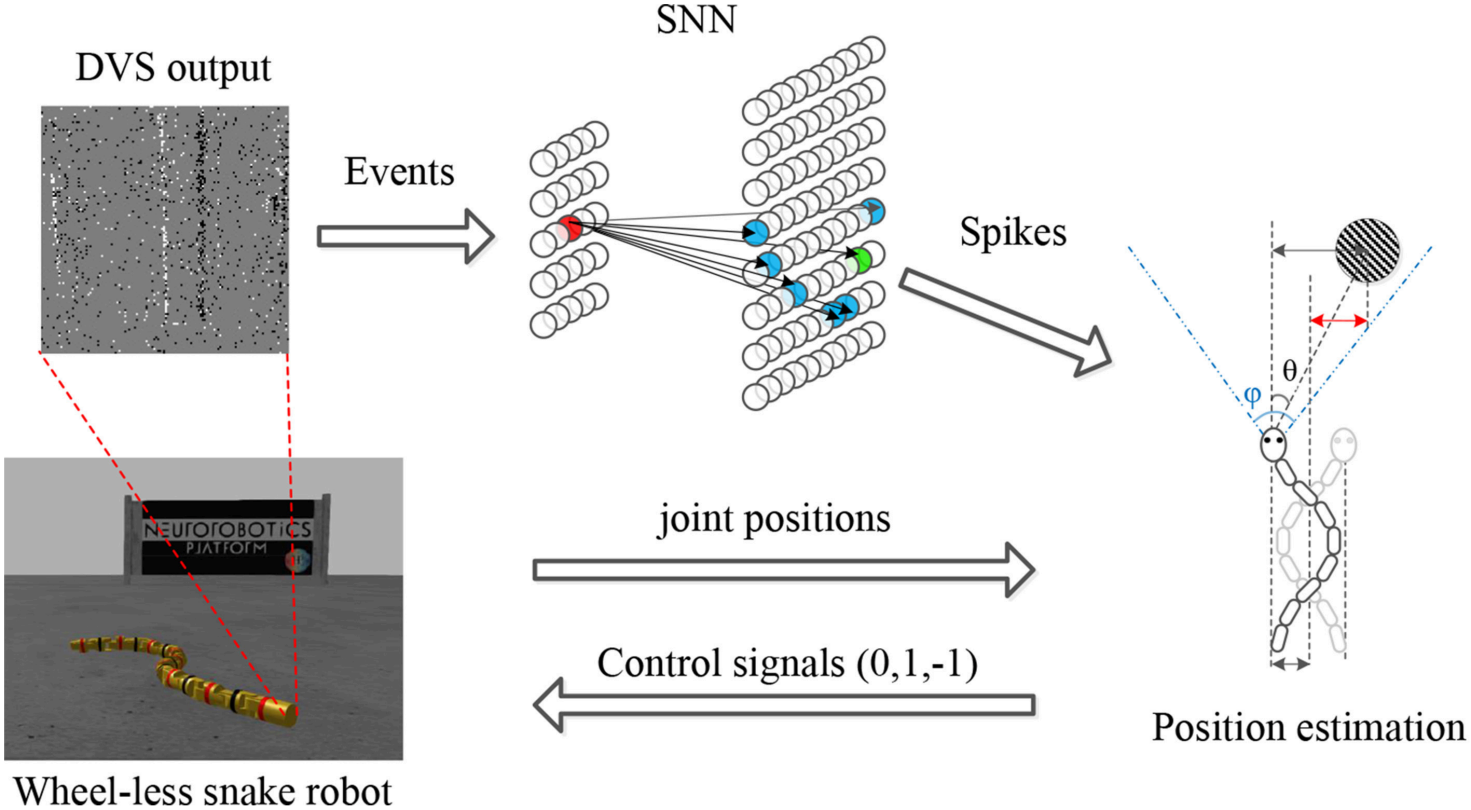
Tracking framework. The asynchronous events derived from the DVS are processed by an SNN designed for object detecting. A neuron (in red) in the input layer fixedly connects some neurons (in blue) according to the principle of the Hough transform. Some neuron (in green) excites when the membrane potential exceeds a threshold. By combining the joint position information obtained from joint encoders and the target position in camera, the relative position of the target (in red) is estimated to generate the control signals. Finally, the wheel-less snake robot approaches the target under a slithering gait.

Target tracking is a typical instance of the autonomous locomotion control. Therefore, tracking framework consists of three components: sensing, planning and acting, which are also the essential components of an autonomous system (Ponte et al., 2014). More concretely, the proposed framework for pipe-like object tracking on a wheel-less snake robot is composed of 4 constituents, as shown in Figure 1. (1) Sensor. The DVS camera observes the environment and generates asynchronous events as the input of the SNN. For an event *e*(*t, x, y, p*), (*x, y*) indicates which neuron receives this input spike, *t* is the time when the spiking neuron receives this input spike, and *p* (±1) defines the voltage of this input spike. Meanwhile, the joint encoder records the position of each joint of the snake robot in a short time period. (2) Spiking Neural Network. A two-layer SNN was designed for object detecting. A neuron in the input layer fixedly connected some neurons in the output layer according to the principle of Hough Transform. The asynchronous events were fed into the input layer of the SNN and impacted the neurons in the output layer by propagating the spikes on the synapses. Once any output neuron excited, a output spike was generated which means a successful detection of the target. (3) Decision maker. It is a non-spiking part. The joint position information obtained from the joint encoders as well as the target position obtained from the SNN were fused to estimate the relative position of target and generate control signals. The function of control signals we used is essentially the same as a Bang-Bang controller. (4) CPG controller. This part is a built-in controller of the wheel-less snake robot that converts the control signals into the parameters of the Central Pattern Generator (CPG) to maintain or adjust the specific locomotion gait.

### 2.2. Model of Spiking Neural Network

Compared to traditional artificial neural networks, spiking neural networks are more similar to the biological brains due to incorporating the concepts of spike-driven synaptic dynamics and temporal dynamics. Temporal dynamics mean the membrane potential of spiking neurons changes spontaneously over time and spike-driven synaptic dynamics describe the information propagation on synapses.

For the neuron model, the most popular one is the Leaky Integrate-and-Fire (LIF) model (Burkitt, 2006), which can be described by the following linear differential equation:

(1) $$\begin{array}{l} \tau_{m}\frac{dv}{dt}=-v\left(t\right)+RI\left(t\right). \end{array}$$

where *v*(*t*) represents the membrane potential at time t, τ_*m*_ is the time constant and *R* is the membrane resistance. A LIF neuron is a simple resistor-capacitor circuit where the leakage term $\frac{-v\left(t\right)}{\tau_{m}}$ is due to the resistor and the integration term $\frac{RI\left(t\right)}{\tau_{m}}$ is due to the capacitor that is parallel to the resistor.

The behavior of a LIF neuron can be depicted as follows. (1) A spiking input causes an increase of the Membrane Potential (MP) of the neuron. (2) In the meantime, the MP always spontaneously decays at a fixed rate. (3) When the MP exceeds the threshold, a spike is generated as an output. Then, the MP of the fired neuron is reset to zero so that the neuron enters a refractory period, during which the MP remains zero and all input spikes are ignored. Because of the similarity between the dynamics of the LIF neuron and the voting process of shape detection based on the Hough transform, the SNN composed of LIF neurons is particularly well adapted for detecting line and circle.

In this work, we designed two SNNs composed of the LIF neurons for line detection and circle detection, respectively. All the SNNs contain a two-layered topological structure, including an input layer and an output layer, as shown in Figure 1. The input neurons obtained the events from DVS and duplicated it immediately. The output neurons integrated the spikes and excited when they received enough spikes, which are extended LIF neurons with both a positive threshold and a negative threshold. Each input neuron permanently connected some output neurons according to the equation of specific shape. The membrane potential dynamics of extended LIF neurons was described as Algorithm 1.

**Algorithm 1:** The membrane potential updating of an extended LIF neuron (λ is the fixed decay rate, *v* is the MP)

1: **for** input spike *s*_*i*_ with polarity *p*_*i*_ at *t*_*i*_ **do**
2: *v*_*i*_ ← *sign*(*v*_*i*−1_)·max(|*v*_*i*−1_|−λ·(*t*_*i*_−*t*_*i*−1_), 0)
3: *v*_*i*_ ← *v*_*i*_+*p*_*i*_
4: **if** |*v*_*i*_|≥*v*_*th*_ **then**
5: Generate output spike δ = *sign*(*v*_*i*_) at *t*_*i*_
6: Reset all connected neurons
7: *v*_*i*_ ← 0
8: **end if**
9: **end for**

#### 2.2.1. Line Equation

According to the Hough transform, we assume $\vec{n}=\left(\sin\theta ,\cos\theta \right)$ as the normal vector perpendicular to the line *L* and ρ as the normal distance from the line to the origin. Hence, a point $\vec{p}=\left(x,y\right)$ on the line L can be formulated as the equation:

(2) $$\begin{array}{l} \rho =\vec{n}\cdot \vec{p}=x\sin\theta +y\cos\theta \;, \end{array}$$

which maps each point (*x, y*) from Cartesian coordinate into parameter space of (θ, ρ) as a sinusoidal curves.

**Figure 2:**
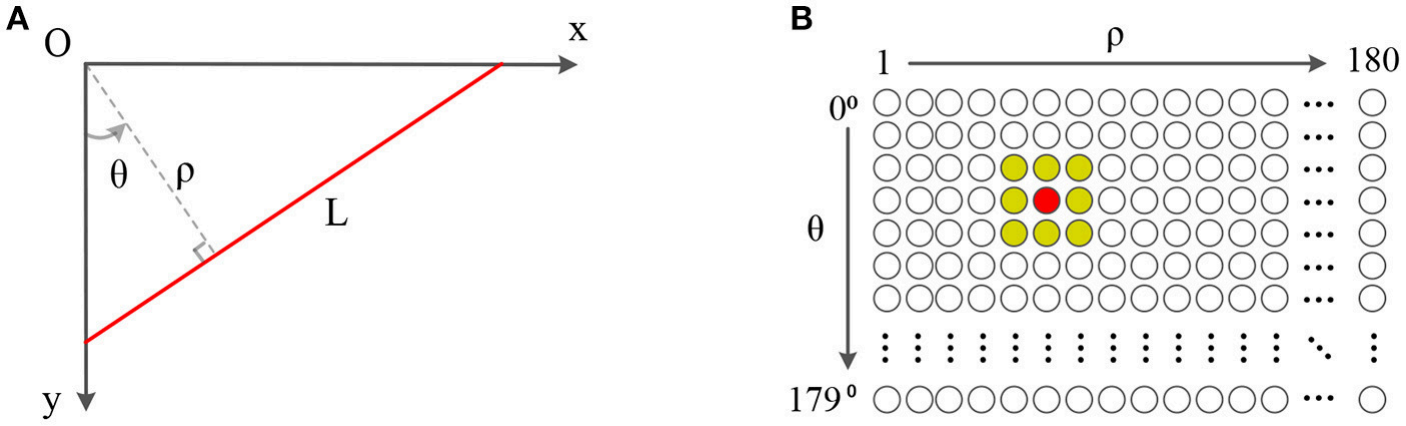
Panel **(A)** is a line in the Cartesian coordinate. Panel **(B)** is a 180 × 180 SNN which θ is limited in [0°, 180°) while ρ is limited in (0, 180]. The central neuron in red corresponds to the line *L* in **(A)**, which is connected to all neurons in its neighbor for local inhibition.

As shown in Figure 2, the SNN corresponding to the parameter space of (θ, ρ) is built up, which consists of 180 × 180 spiking neurons. The first dimension of the SNN represents the angle θ and the second dimension is the distance ρ. In this example, the range of θ is [0°, 180°) with 1° resolution and the range of ρ is (0, 180] pixels (180 approximately equals to the diagonal distance of the view field of a DVS128) with 1-pixel resolution. Each neuron of the SNN represents a line with (θ, ρ) in the parameter space.

#### 2.2.2. Circle Equation

As we all know, a point *p* = (*x, y*) on a circle C can be described by the following standard equation:

(3) $$\begin{array}{l} {\left(x-a\right)}^{2}+{\left(y-b\right)}^{2}=r^{2}\;, \end{array}$$

where (*a, b*) is the coordinate of the center of the detected circle.

An SNN for circle detection is a three-dimensional parameter space of (*a, b, r*), which consists of 128 × 128 × 64 spiking neurons. The first two dimensions of the SNN represent the position of a circle center in the horizontal direction and the vertical direction, respectively, while the third dimension is the radius of a circle. In this case, the resolutions in all of the three dimensions are 1 pixel. Each neuron of the SNN represents a circle with (*a, b, r*) in the parameter space.

#### 2.2.3. Lateral Inhibition

The local lateral inhibition, which is a nature of biological neurons, was applied to suppress the noise in this work. Every spiking neuron was connected to its adjacent neurons. Once a shape was detected, a spiking neuron would excite and all the spiking neurons connected would be inhibited. In order to make a trade-off between the result of noise suppress and the computation cost, we select a 3 × 3 window as the range of local lateral inhibition. This means that neurons in the 8-adjacent of the fired neuron are reset. When a larger inhibition range is used, the target would be detected less often in approximately the same region, but more reset operations need to be done—otherwise, the reverse.

### 2.3. Pipe Detection

For pipe detection, the different poses of a vision sensor will result in different shapes of a pipe in the image plane, a pair of parallel lines or a circle. The circle can be detected directly by an SNN, while a strategy needs to be proposed for recognizing two parallel lines.

The edges on both sides of the pipe body can be detected as two parallel lines. In an indoor environment, the change of brightness is opposite on both sides of the pipe while the DVS camera moves perpendicular to the pipe. The polarity of the events on one edge of the pipe is positive while that on another edge is negative. Once the DVS camera moves to the opposite direction, the polarity of the events on two edges would reverse. The two lines with opposite polarity can be considered as the body of a pipe. Furthermore, this pair of parallel lines should appear at the maximum frequency which is equivalent to the highest fired rate of the spiking neuron. Therefore, three conditions to judge whether a pipe appears are listed below:

- The polarities of the two lines are opposite.
- The two lines are parallel or the difference of the angle is tiny.
- The pair of parallel lines appears at the maximum frequency in a short time period.

In this work, we tested all the detected lines in each time slot and only one pair of lines satisfying the above conditions would be found out. The target can be detected by Algorithm 2 and represented as a 4-tuple *P*(*t*, θ, *w, pos*), where *t* is the timestamp, θ is the angle of the pipe, the *w* is the width of the pipe and the *pos* is the offset in pixel which is relative to the left side of the field of view.

**Algorithm 2:** Event-based pipe detecting in the SNN

1: **for** each time slot **do**
2: **for** event *e*_*i*_ = (*t*_*i*_, *x*_*i*_, *y*_*i*_, *p*_*i*_) **do**
3: **for** every angle θ_*j*_ in SNN **do**
4: Calculate distance ρ_θ_*j*__ = *argmin* |ρ−*x*_*i*_sinθ_*j*_−*y*_*i*_cosθ_*j*_|
5: Update neuron *N*(θ_*j*_, ρ_θ_*j*__) at *t*_*i*_ with polarity *p*_*i*_
6: **if** a neuron fires **then**
7: Store the output spike
8: **end if**
9: **end for**
10: **end for**
11: Find out a pair of output spikes satisfying the judgment conditions
12: **if** a pipe exits **then**
13: Output the pipe *P*(*t*, θ, *w, pos*)
14: **end if**
15: **end for**

### 2.4. Tracking

#### 2.4.1. Motion Analysis of Snake Robot

Due to the fact that the DVS was mounted on the head of the snake robot, the horizontal offset and the orientation of the head should be known for obtaining precise tracking performance. Therefore, we collected the data from the joint encoder installed on the head and recorded the pose of the head, which includes the trajectory and the rotation in the simulation environment. Then, the horizontal offset and the orientation were analyzed by using FFT. After applying the head orientation compensation approach, the head of the snake robot kept always looking forward along the moving direction, which is a benefit for simplifying the model to estimate the relative position of the target. On the other hand, the horizontal offset was estimated based on the head joint position. This idea is derived from the observation that the rotation of the joints is the essential driving force of the wheel-less snake robot.

For tracking tasks, our snake robot moved under a slithering gait, which provides the most stable pose of camera. The horizontal offset of DVS was fitted by FFT. In the meantime, we reconstructed the horizontal trajectory of the head module by integrating the head joint position θ. In fact, the head joint position and the horizontal trajectory are both the periodic signal, which can be formulated as Equation (4) and Equation (6), respectively. Wu and Ma (2010) indicated that these two signals have the same form but different amplitude and phase; however, it was unable to give the offset of the head module in real time. Therefore, considering the joint rotation is the main driving force and reason to the motion of wheel-less snake robots, the head joint position was regarded as the argument of the horizontal trajectory. By tuning the phase and the amplitude, we got the offset of the DVS from the head joint position according to the Equation (6). Further, the situation of turning was approximately treated as that of moving straight.

(4) $$\begin{array}{lll} \theta & = & a\cdot sin\left(\omega \cdot t+\phi \right) \end{array}$$

(5) $$\begin{array}{l} \theta_{tuned}=\alpha \;\cdot \;a\;\cdot \;sin[(\omega \;\cdot \;t+\phi )+\phi_{pos}]\\ \;=\alpha \;\cdot \;(\theta \;\cdot \;cos\phi_{pos}+\theta^{\prime }\;\cdot \;sin\phi_{pos})\;. \end{array}$$

(6) $$\begin{array}{l} Position=A\;\cdot \;sin(\theta_{tuned})=A\;\cdot \;sin[\alpha \cdot (\theta \;\cdot \;cos\phi_{pos}\\ \;+\theta^{\prime }\;\cdot \;sin\phi_{pos})]\;. \end{array}$$

where *A* is the amplitude of the swing which is perpendicular to the direction of motion, α is the ratio of the 90 degrees to the *a*, θ is the value of the joint encoder, θ′ is the joint velocity which is also the first-order derivative of θ and ϕ_*pos*_ is the phase difference. In our case, the tuple (*A*, α, ϕ) is (–0.124, 1.000, 0.000) for V-REP and (1.400, 2.571, 0.908) for NRP, respectively. These parameters are different in V-REP and NRP because the joint controllers and environment parameters in these two simulators are a little bit different.

#### 2.4.2. Relative Position Estimation

By using a distance sensor, such as an ultrasonic sensor, an IR sensor, etc., we can actively measure the relative distance between the snake robot and target. However, a time-of-flight distance sensor is usually directed, which means more sensors need to be installed in the limited space of the snake robot, especially when the position of the target is unknown. It is plausible to the actual snake robot as well. Besides that, when using a DVS along with distance sensors, we must ensure the consistency of different sensor measurements, that is, to make sure the measured data represent the same object. Therefore, aiming to use fewer sensors, we detected the target and estimated depth simultaneously by using a single DVS sensor.

**Figure 3:**
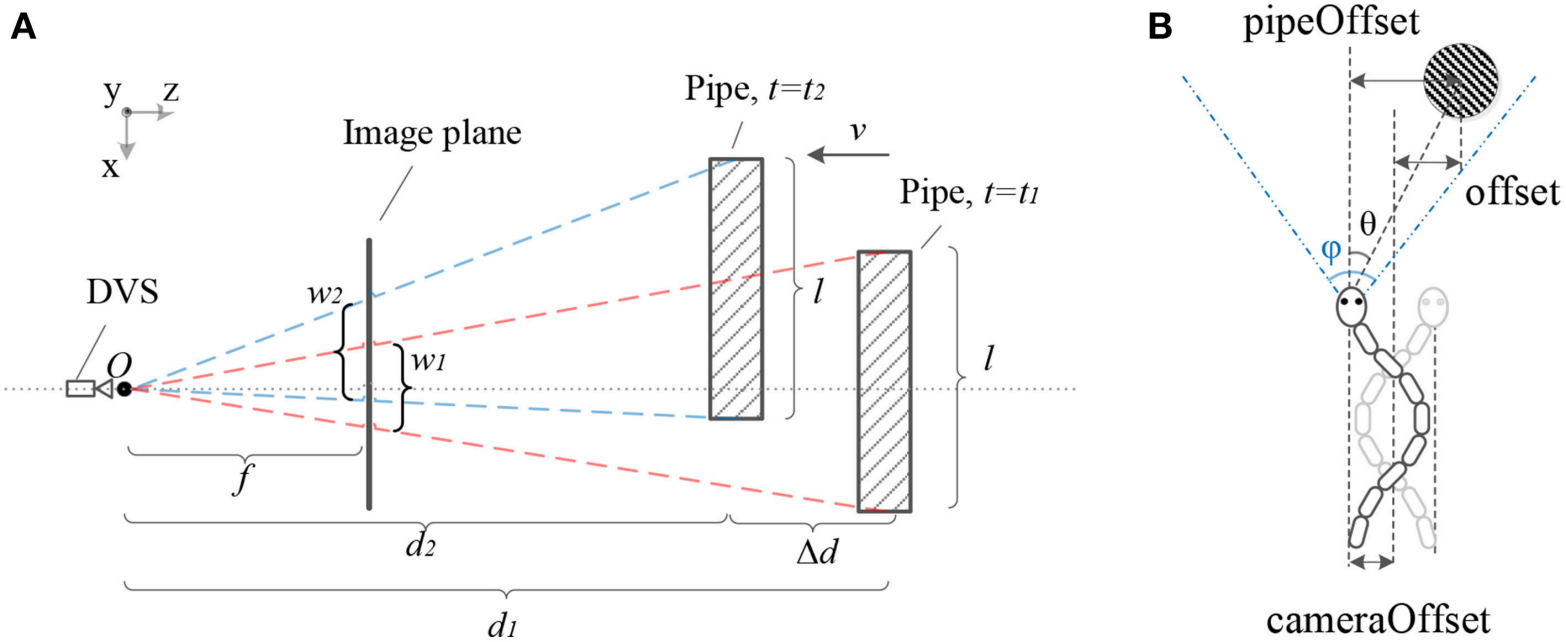
Panel **(A)** shows the geometric relationship between the DVS camera and the target pipe. When *t* = *t*_1_ the pipe is on the far position from camera. After the snake robot moves at an approximately constant velocity *v*, the pipe is on the near position when *t* = *t*_2_. Panel **(B)** shows the geometric relationship among the pipe offset in the image plane, camera offset and actual offset while the snake-like robot moves forwards with a periodic swing.

Once the target is detected by the SNN, we can estimate the offset of the target in the horizontal plane and the forward relative distance between the DVS camera and the target. As shown in Figure 3A, in a time period Δ*t* = *t*_2_−*t*_1_, there is a functional relation between the decrease of the distance Δ*d* = |*d*_2_−*d*_1_| on the z-axis and the increase of the visible width of the target Δ*w* = |*w*_2_−*w*_1_|. Besides that, the distance *d* is always inversely proportional to the width of the target *w*, the scale factor is the focus length *f* multiplying the actual width *l*. Moreover, we can reasonably assume that the snake robot moves forward at an approximately constant speed since that is very small. Hence, the Δ*d* can be estimated by multiplying the elapsed time and the speed of the snake robot. In summary, we can calculate the distance *d*_2_ depending on the displacement in a time period and the change of the target visible width according to the following equations.

(7) $$\begin{array}{l} \Delta d=|d_{2}-d_{1}|=v\cdot \Delta t\;, \end{array}$$

(8) $$\begin{array}{l} d=f\cdot l\cdot w^{-1}\;, \end{array}$$

(9) $$\begin{array}{l} d_{2}=\frac{f\cdot l}{w_{2}}=\frac{w_{1}\cdot v\cdot \Delta t}{\Delta w}\;. \end{array}$$

The snake-like robot moves slowly and the Δ*t* and the Δ*w* between two consecutive output spikes are tiny. Therefore, the error of distance calculated by the Equation (9) is remarkable. To reduce the error, two discrete output spikes are selected for distance estimating and the interval is 10 spikes in this paper.

Assuming that the position of the moving snake robot and the target pipe satisfies the relationship shown in Figure 3B, the snake robot swings along the central line while it is moving forwards. Hence, in order to obtain the offset between the central line and the target, we calculated two kinds of offset, the *pipeOffset* and the *cameraOffset*, respectively. Further, we proposed the method to estimate the pipe position for tracking as shown in Algorithm 3. We calculated the *pipeOffset* according to the ration of tanθ to $\tan\frac{\phi }{2}$ (ϕ is the FOV of DVS). Then we estimated *cameraOffset* according to the Equation (6).

**Algorithm 3:** Position estimation and pipe tracking

1: **for** pipe *P*_*i*_(*t*_*i*_, θ_*i*_, *w*_*i*_, *pos*_*i*_) **do**
2: **if** *l* = *Size*(*pipe*) is known **then**
3: Calculate the distance $d=f\cdot l\cdot {w_{i}}^{-1}$
4: **else**
5: **if** *i*≥10 **then**
6: Calculate the distance $d=v\cdot \left(t_{i}-t_{i-10}\right)\cdot \frac{w_{i-10}}{w_{i}-w_{i-10}}$
7: **else**
8: Set the distance *d* to 0
9: **end if**
10: **end if**
11: Calculate the offset of the pipe in DVS, $pipeOffset=d\cdot \frac{|{\text{pos}}_{i}-64|\cdot \tan\frac{\phi }{2}}{64}$
12: Estimate the offset of the camera *cameraOffset*
13: Calculate the actual offset *offset* = *pipeOffset*+*cameraOffset*
14: Generate control parameter *C* = *Sign*(*offset*), where −1 means turning left, +1 means turning right and 0 means going straight
15: **end for**

### 2.5. GPU Acceleration

For artificial SNNs, the neurons will update their states only when input spikes arrive asynchronously. When SNNs are implemented on CPU or neuromorphic chips, the neurons can update asynchronously as well. However, the general CPU is unable to deal with the large quantity of communication between neurons and real-time state updating of neurons. The neuromorphic simulators and chips still have some drawbacks on running a large SNN. Therefore, we tried to accelerate our SNN by using a GPU. By providing a uniform clock, the neurons could update synchronously in a short time period, such as frame-based image processing.

## 3. Experiments

Our tracking framework was evaluated both in NRP and V-REP, which is a robot-brain simulator and a robotics simulator, respectively. To begin with, we reconstructed the trajectory of the head module of the snake robot for obtaining the camera offset perpendicular to the forward direction. Then, two scenes were built. One of them only had a standing pipe while another one contained a lying pipe. Finally, experiments were conducted on the aforementioned scenes. Meanwhile, the SNN was performed on a CPU by only using a single thread and a CUDA GPU, respectively.

### 3.1. Simulation Environments

**Figure 4:**
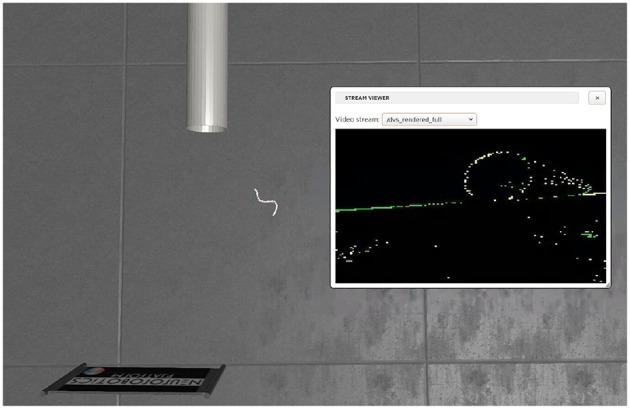
The simulation environment in NRP, which includes a hollow pipe and a snake robot with a DVS.

The Neuromorphic Platform (NRP) (Roehrbein et al., 2016; Falotico et al., 2017) is an integrated simulation platform to facilitate a direct link between robotics and neuroscience. In its Gazebo-based world simulator, we built a modular wheel-less snake robot and a simple environment in which there was only one pipe, as shown in Figure 4. we built two SNNs as well, respectively for pipe body detection and pipe entry detection. By using the Robot Operating System (ROS) as a communicating middle-ware, our snake robot and the SNNs could exchange data and commands through ROS topics. While V-REP (Rohmer et al., 2013) is a simulator only for robotics in which we implemented SNNs outsides and connected the snake robot and SNNs by utilizing Remote APIs. Although the NRP and the V-REP are both robotics simulators, models of the snake robot are a little bit different in the number of modules, the controller parameters and so on.

### 3.2. Results and Discussions

We conducted experiments in V-REP and NRP, respectively, considering four situations, which are (1) tracking pipe body on the left side of the snake robot, (2) tracking pipe body on the right side of the snake robot, (3) tracking pipe entry on the left side of the snake robot and (4) tracking pipe entry on the right side of the snake robot.

**Figure 5:**
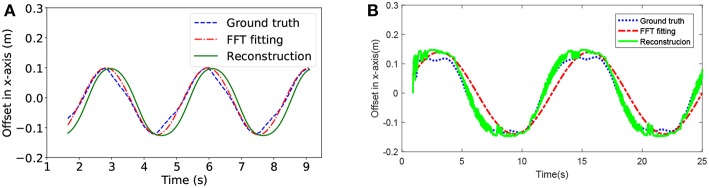
The horizontal offset estimation for the head module of the snake robot while it is moving forwards. Panel **(A)** is from V-REP, Panel **(B)** is from NRP.

First, we fitted the camera offset by using the head joint position. As shown in Figure 5, the blue curve is an approximately sine curve that represents the actual offset of the DVS camera, the red one is the offset analyzed by FFT and the green one is the reconstructed offset. We not only estimated the offset according to the Equation (6), but also applied a mean filter on the timeline to smooth the estimation result. Therefore, the horizontal offset of the DVS camera was directly obtained in real time under a low estimation error.

**Figure 6:**
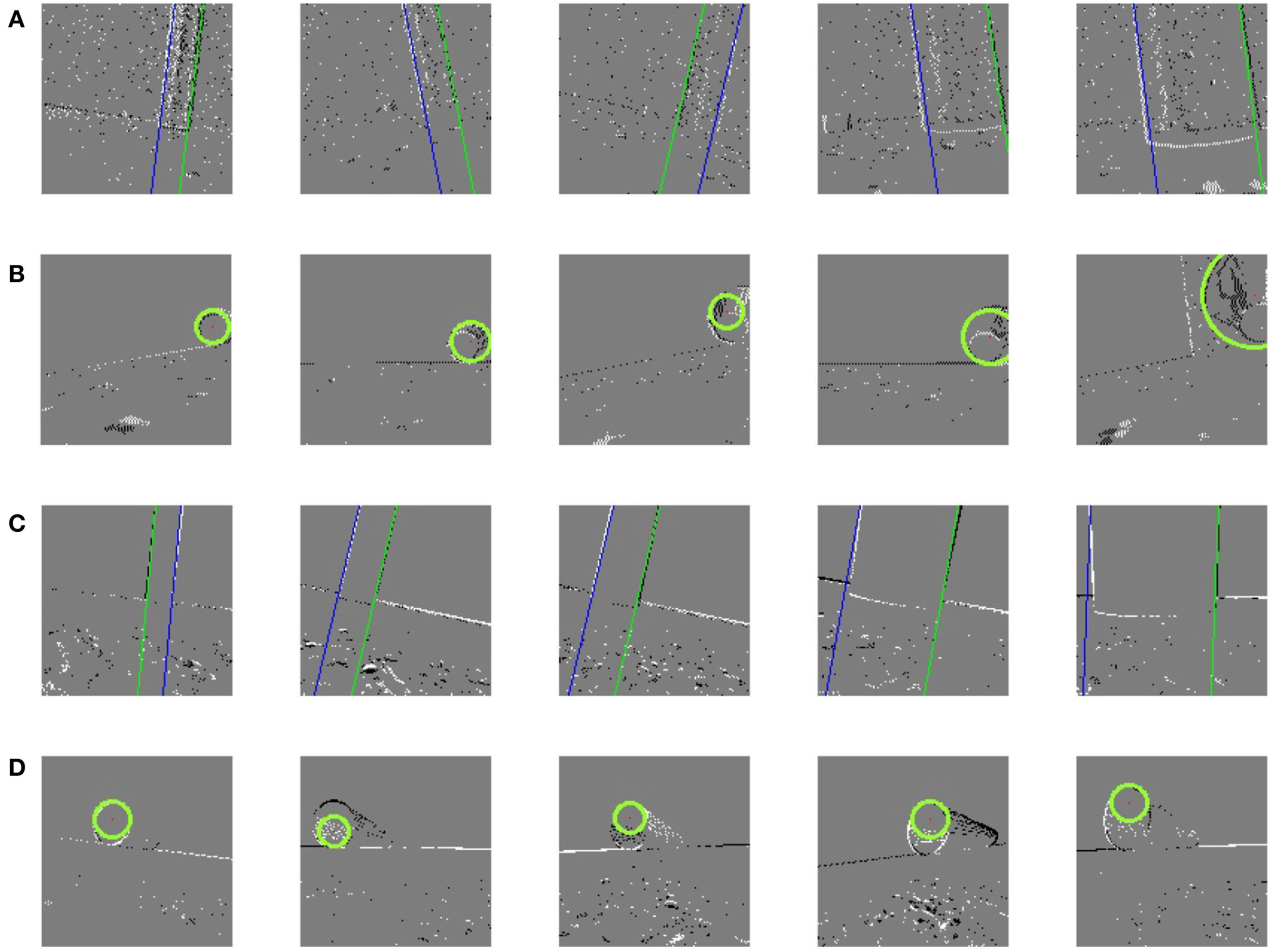
Results of pipe detection. **(A)** Detected pipe body in V-REP. **(B)** Detected pipe entry in V-REP. **(C)** Detected pipe body in NRP. **(D)** Detected pipe entry in NRP.

Then, for the pipe detection, we put a hollow pipe at different perspectives of the DVS camera. The snake robot always started moving from the same initial position for each situation, but the initial position in V-REP is different from that in NRP. During the detecting procedure, the event sequences generated by the DVS were fed into the vision SNN. Events were asynchronously processed, but we only detected pipe once in each time slot according to the method discussed in section 2.3, and the image and the position of the pipe were recorded and a part of them was shown in Figure 6. The standing pipe was represented by a blue and a green line and the lying pipe was represented by a green circle. The precision of the standing pipe detection is higher than the lying pipe detection because of the limitation of the network size, especially for circle detection. More neurons means greater ability to recognize the much finer structure of the target. Additionally, it suffered from worse precision when the snake robot got close to the target, especially the lying pipe. At the beginning, the circle looks dense and easily distinguished, however it looks noisier because more details of the target which generated noise spikes were seen. If we increased the firing threshold of the membrane potential to increase the precision of target detection, however, the firing rate of output neurons would reduce so significantly that there were not enough output spikes generated.

**Figure 7:**
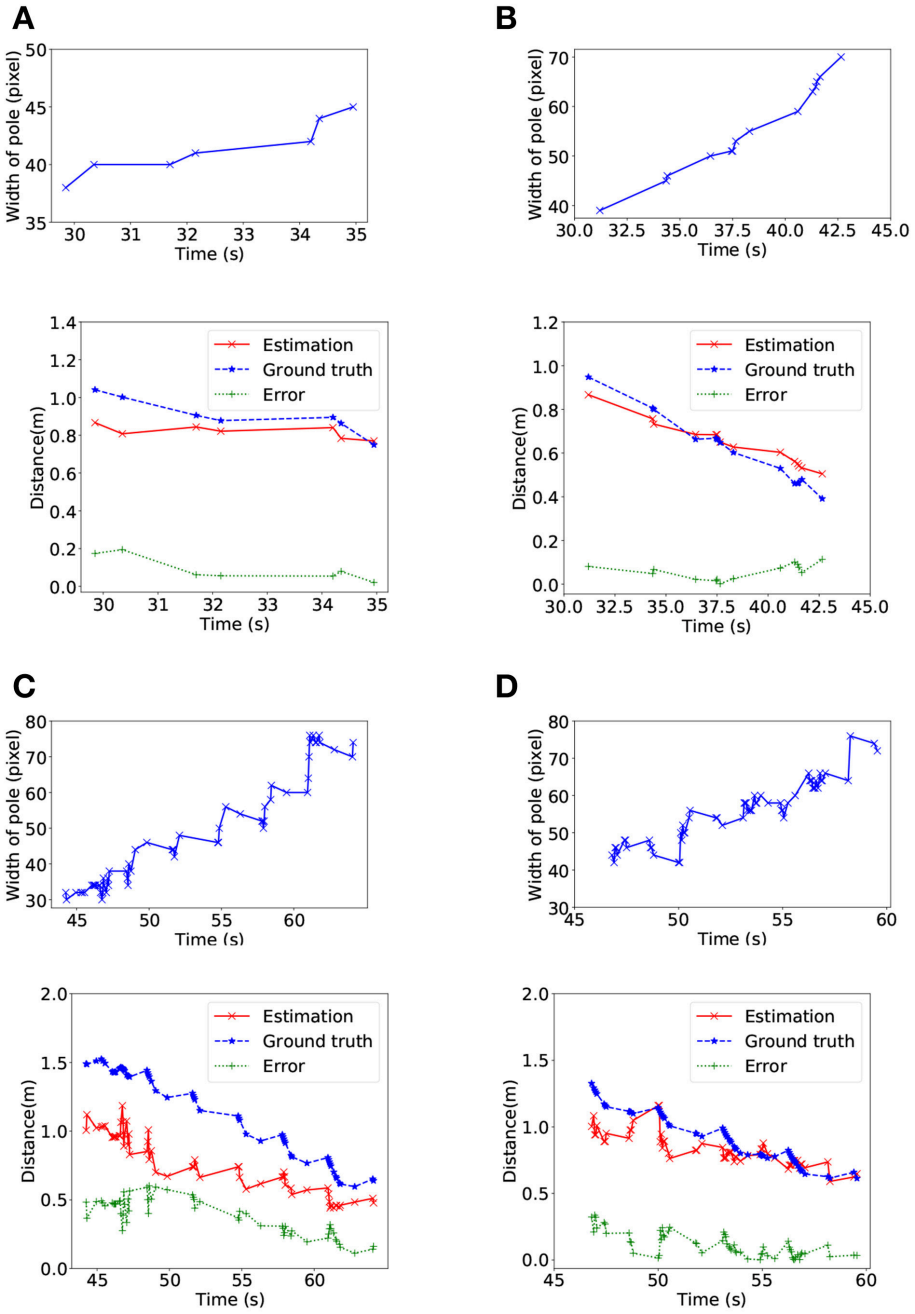
Relative position estimating in V-REP. For each case, the upper line shows the increase of the width of the target pipe in pixels and the lower line shows the distance between the head of the snake robot and the target pipe. In **(A)** and **(C)**, the target pipe is on the left side of the snake robot, and the target pipe is on the right side in **(B)** and **(D)**.

After that, the relative distance between the DVS camera and the target pipe was estimated by using Algorithm 3. As the snake robot moving toward the pipe, the width of the pipe increased in the image plane. As shown in Figures 7A,B, the relative distance decreased when the width of pipe increased and the average error was around 0.1 m. Nevertheless, the error of Figures 7C,D were much higher than for standing pipe tracking. The reason is that the firing rate was much higher and the difference of width was smaller than parallel lines detection when we detected the circle. Additionally, in all cases, larger error also occurred at the early time period of the simulating experiment. We only evaluated the precision on the data derived from V-REP because we assumed the size of targets was known in NRP to avoid introducing too much error.

**Figure 8:**
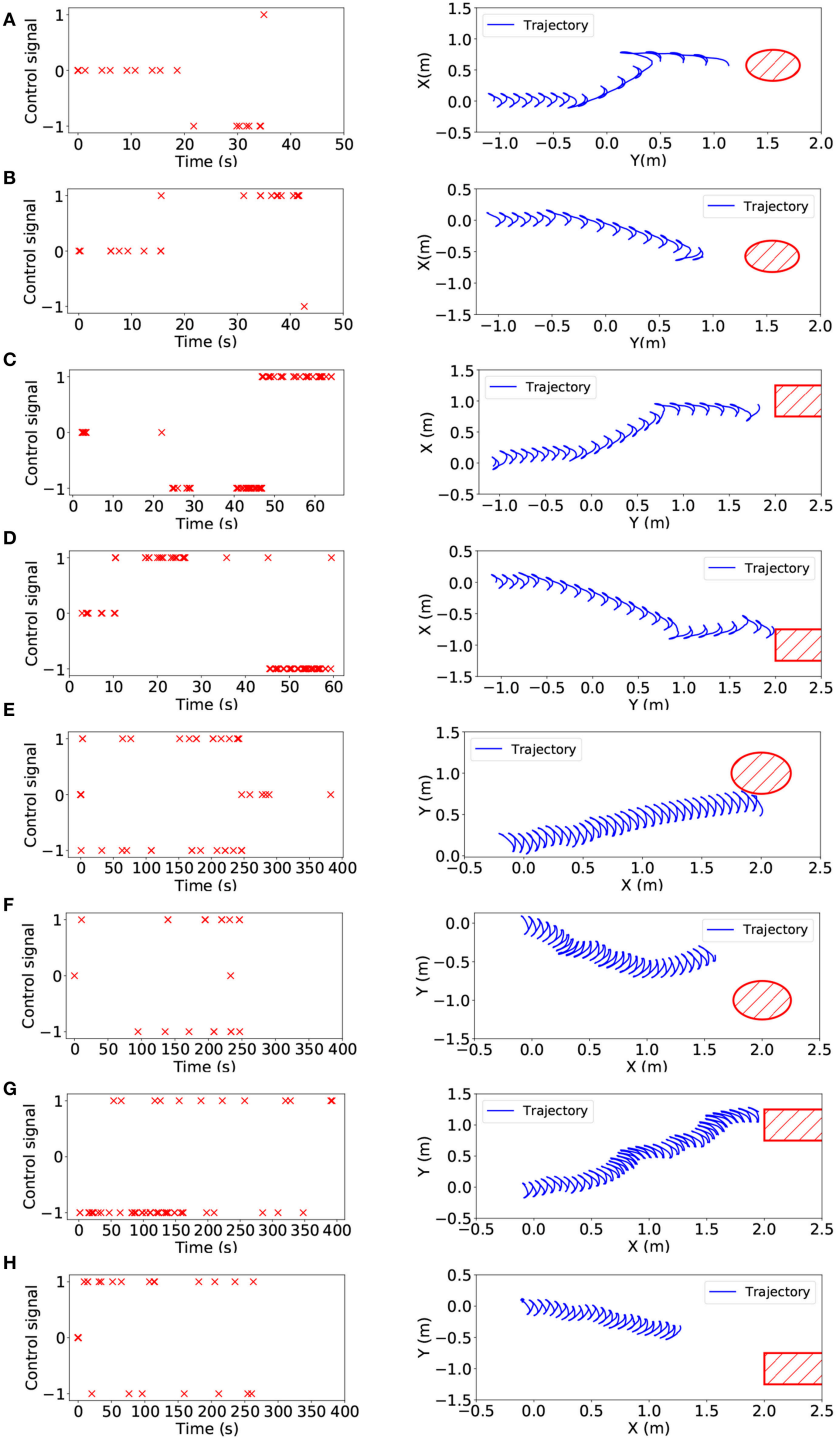
Left: Control signals of turning left(–1), turning right(1) and going straight(0) for the snake-like robot. Right: The overhead view of the trajectory of the head module while tracking. From **(A–H)**, eight experiment results are shown which are left-side standing pipe tracking in V-REP, right-side standing pipe tracking in V-REP, left-side lying pipe tracking in V-REP, right-side lying pipe tracking in V-REP, left-side standing pipe tracking in NRP, right-side standing pipe tracking in NRP, left-side lying pipe tracking in NRP, right-side lying pipe tracking in NRP.

Furthermore, we estimated the actual offset of the target pipe and generated control signals so that our snake robot was able to achieve pipe tracking, as shown in Figure 8. Figures 8A–D showed the final trajectory of the snake robot for tracking in V-REP, the shapes in red were the actual position of the target pipe. Then, Figures 8E–H showed the final trajectory of the snake robot for tracking in NRP. All the results demonstrated that our tracking framework based on SNN was valid and effective. The snake robot was able to find the target pipe and approach it by performing a series of motion, including turning left, turning right and go straight. The trajectories shown in Figure 8 were not representative of a smooth curve because of the swing of the snake robot, but the trend of motion is still correct. Another feature about the curves was that there were several obvious turning points in V-REP but not in NRP.

Finally, we compared the performance of the proposed SNNs on CPU and GPU, respectively. As shown in Table 1, the GPU was able to accelerate the detection procedure for each case, especially for lying pipe detection. Due to the three-dimensional parameter space for lying pipe detection, the GPU could achieve higher speedup than standing pipe detection. Moreover, in cases 3 and 4, the pipe detection could be performed in real time by using the GPU. In addition, the simulating experiments conduced in NRP showed higher frame rate than that of V-REP, especially when the GPU was utilized. The possible reason is that the V-REP spent more time in data transferring between the snake robot and the SNN.

**Table 1:** The frame rate of pipe tracking on CPU and on GPU, respectively.

**Case**	**Hardware**	**CPU(fps)**	**GPU(fps)**	**Speedup**
Standing pipe in V-REP	Intel i7-5500U/Nvidia Geforce 940 m	0.21	1.71	8.14
Lying pipe in V-REP	Intel i7-5500U/Nvidia Geforce 940 m	0.33	3.22	9.76
Standing pipe in NRP	Intel i7-4770/Nvidia Geforce GTX 645	1.63	40.74	24.99
Lying pipe in NRP	Intel i7-4770/Nvidia Geforce GTX 645	0.36	22.35	62.08

## 4. Conclusion

In this work, we proposed a pipe-like object detecting and tracking approach by combining the DVS and SNNs, and successfully performed on a wheel-less snake robot. The target pipe was detected by dealing with the asynchronous address-event stream obtained from a DVS. Then, an autonomous tracking method was present according to the relative position between the snake robot and the target. Furthermore, the performances of the proposed SNNs were estimated on CPU and GPU. The experiments demonstrated the efficacy of our tracking approach based on SNNs and showed the practicality and accuracy of the autonomous tracking method. Comparing the performances of our SNN model on CPUs and on GPUs, respectively, the SNN model showed the best performance on a GPU while is displayed the highest precision on a CPU. However, there are still some drawbacks to our approach. The prime one is that the performance of tracking is sensitive to the noise and the error in detection and position estimation.

## Author Contributions

ZJ and AK brought up the core concept and architecture of this manuscript. ZJ and ZB designed the experiments. ZJ, ZB, and KH wrote the paper.

### Conflict of Interest Statement

The authors declare that the research was conducted in the absence of any commercial or financial relationships that could be construed as a potential conflict of interest.
